# *Current Issues in Molecular Biology* Journal Enters a New Era

**DOI:** 10.3390/cimb43010031

**Published:** 2021-06-18

**Authors:** Rafael Franco

**Affiliations:** 1 Department Biochemistry and Molecular Biomedicine, University of Barcelona, 08028 Barcelona, Spain; rfranco123@gmail.com or rfranco@ub.edu; 2 School of Chemistry, University of Barcelona, 08028 Barcelona, Spain; 3 Centro de Investigación Biomédica en Red Enfermedades Neurodegenerativas (CiberNed), Network Center, Neurodegenerative Diseases, Spanish National Health Institute Carlos III, 28031 Madrid, Spain

## Introduction

*Current Issues in Molecular Biology* (*CIMB*) (https://www.mdpi.com/journal/cimb) (accessed on 17 June 2021) was born in 1999, at the end of a century in which molecular biology became associated with the structure of DNA and, by extension to DNA replication, transcription into RNA, and the translation of mRNA into proteins [1,2]. Despite seminal papers on the regulation of transcription, such as the ones reporting the discovery of the lactose operon [3,4,5,6,7,8,9], so-called transcription factors came into vogue later. It is worth reading the summary of the first paper in the inaugural issue written by biotechnology consultant Robert C. Tait, entitled “The Application of Molecular Biology” [10]. The comments are still valid today but the perspective has been dramatically changed as Molecular Biology is no longer restricted to the study of the role of nucleic acids and the development of techniques related to assessing the sequence and structure of nucleic acids. Molecular Biology is now a holistic discipline in the realm of life on Earth.

## Advancement versus Data Generation/Collection

The current Century started with high hopes due to the development of technologies to quickly obtain myriad of data that are deposited in multiple databases. It is possible to sequence any genome, to know the concentration of hundreds of metabolites in a given cell/system, to “decipher” the transcriptome of a given cell, etc. However, the challenge is to convert information into knowledge and *CIMB* cares about this. The journal will give priority to certain types of papers and among others, one example would be a paper information from databases and converting it into useful information. Other journals are expected to have a similar goal of changing the “forward flight” trend, that is, producing more and more data without addressing what the new data means. In other words, data collection is not an objective per se in the mind of the *CIMB* editorial board members. Take, for instance, the generation of countless SARS-CoV-2 sequences. On the one hand, the generation of said data has not been coordinated to be able to carry it out for a specific purpose or to contrast any particular hypothesis. On the other hand, and what is more worrying, no attempt has been made to perform an RNAseq of patient samples to try to find out which factor(s) in the host influence the severity of the disease. Paraphrasing the discoverer of the fourth phase of water, Gerald H. Pollack ([11]), never in history has so much money allocated to science provided so little useful results. In fact, whereas the technological advances have been impressive, the actual advances in the intricacies of our biological processes have been very limited and never reaching the level of the discovery of the double helix of DNA. This century should change the trend and journals have the responsibility to contribute to creating a climate that fosters scientific advancement. Due to the way the fourth state of water impacts on the view of life at the molecular level, it is a magnificent tool for revisiting current data and emitting novel hypotheses for testing using suitable resources. Sharma and Pollack have recently hypothesized that the fourth phase of water and the so-called “exclusion zone” must be taken into account in revisiting fat’s role in cell membrane physiology and understanding any health-promoting function2 of fats [12].

## Fashionable Proteins and Fashionable Words in Biology

Whereas DNA is a word/abbreviation that is central to biology and must appear in millions of papers, there are words that deserve much less attention. One example is the nuclear factor kappa-light-chain-enhancer of activated B cells or, in brief, NF-κB factor. When it was discovered in the 1980s, it showed promise because it was believed to be expressed in a few cells of the immune system and because it mediated some effects of the human immunodeficiency virus [13,14]. The protein is now considered ubiquitous and has been linked to virtually any scientific topic and/or disease [15,16]. A search for “NF-κB” in google scholar leads to 203,000 hits, 18,200 since 2020. What this means is that it has become so famous that any search using a cell-related work and NF-κB will retrieve some result. Is NF-κB the answer for everything? Is it the Rosetta Stone of biology? It is not likely. In practice, any analysis of big (biological) data will be skewed depending on the literature that includes both studies on NF-κB and just the name of the factor written in any section of the paper. In the papers submitted, *CIMB* will always check for the possibility of biased results and recommend the curation of the data by eliminating any possible bias sources. In addition, any automatic-like tool developed to curate the analysis of data retrieved from databases (or to directly curate databases) will be welcomed in the journal.

## Hints in the New *CIMB* Era

### Articles versus Reviews

The journal has been known for publishing reviews on a wide variety of topics. The new era is more destined to publish original articles describing important advances without forgetting the thematic reviews on hot and/or controversial topics.

It should be noted that the review articles in *CIMB* history have been multidisciplinary and well received by the scientific community. I would like to mention a few published in recent years, all of them well above average in terms of citations according to CiteScore^R^ Scopus. Two of them are devoted to discussing plant issues, one presents the of use of transgenic plants for pharmaceutical biotechnology purposes [17] (citation benchmarking (CB) according to Scopus: 93rd percentile), and another giving insights into diseases derived from replanting [18] (CB: 97th percentile). *CIMB* will continue to welcome valuable articles devoted to any form of life, as well as to the interactions between them: plant/plant, bacteria/plant, bacteria/animal, virus/plant, virus/animal, etc. The 2020 review paper entitled “Cracking the ubiquitin code: The ubiquitin toolbox” [19] has already a field-weighted citation impact of 3.3, and is at the 93rd CB percentile. Very good scores have two excellent articles, one on the molecular mechanisms by which *Salmonella* evades the immune response [20] and another on a factor that controling virus replication becomes a target for antiviral therapy [21]. Last but not least, there is an excellent review on the receptors that control cholesterol homeostasis and, finally, their role in the etiology of cardiovascular disease [22]. A well-rated article discusses adjuvants for DNA vaccines, something that has become a hot topic due to the SARS-CoV-2-driven approval of new types of vaccines [23]. Unexpectedly, the paper entitled “mRNA: A Versatile Molecule for Cancer Vaccines” [24] has a low score despite the fact that it can now be considered to have appeared ahead of its time. On the one hand, immunological-based treatments have proven instrumental in combatting certain types of cancer [25,26,27,28]. On the other hand, it has recently been shown that RNA-based vaccines are the most effective at preventing infection by the virus that causes COVID-19.

The recent appearance of articles in the journal is considered successful. More importantly, *CIMB* has attracted and published papers of significant quality. Checking the last issue, one encounters, among others, papers on:

- the bioinformatics-based assessment of the prognostic value of general transcription factor III in colorectal cancer [29].
- how an edible insect, *Bombyx mori*, protects the liver, thus leaving open the door to finding novel therapeutic molecular approaches to combatting non-alcoholic fatty liver diseases [30].
- the wide variety of anthocyanin levels, whose health benefit potential is well recognized, in selected species and cultivars of berry fruits [31].
- immune responses to retroviral infection in koalas with evidence of underlying mechanisms, suggesting vaccination as a control strategy [32].
- the potential as biomarkers of inflammation and neurotoxicity of apolipoprotein E isoforms [33].
- the whereabouts of adaptation of pancreatic islets function in response to chronic epinephrine exposure [34].
- how carcinoma-infiltrating CD3low Vγ9Vδ1 T Cells may help in anti-tumor responses [35].
- a case-control study assessing the link between hearing impairment and proton pump inhibitors [36]
- a study finding a link between amino acid variants in HLA- DQB1 and -DRB1 allotypes and type 1 diabetes and latent (adult onset) autoimmune diabetes in the Japanese population [37].

In summary, papers are varied, deal with molecular aspects of biology, cover different topics, and contemplate the interface between health and disease, and not only in humans.

### Themes and Configuration of Articles in the New Era

*CIMB* would like to fill a niche that lies between the more molecular and structural aspects of biology and the release of data that cannot be interpreted in terms of providing a biologically sound message. One example is epigenetics, which is a theme that must be covered by the journal. *CIMB* would prefer to know why a single methylation in a specific position in the genome or an acetylation of a specific residue in a histone may lead to biological changes in a given cell rather than comparing the methylation/acetylation patterns found in two different cells or in the same cell in two different conditions.

There is also a need for journals to publish reports of basic science and of applied science, but not necessarily of translational science. *CIMB* welcomes data that may explain the mechanisms of a given pathology but without using any model of the pathology. The lack of models of disease in a paper often impedes its consideration in journals that include the name of a disease or the name of a medical discipline in the title.

Readers are invited to note the diversity already offered in the current issue of the journal. A quick glance at those articles shows the direction the journal is taking. *CIMB* will not forget its origins and will value its reviews as it embarks on a new challenge that consists of caring about the evidence, spending time on accurate data analysis, putting quality before quantity, and aiming to help people better understand the intricacies of life on Earth. Data resulting from preregistered studies are also welcome if the underlying hypothesis is related to a possible advance in understanding (regardless of whether the hypothesis ends up being proven or disproven). Additionally, *CIMB* cares about life and will accordingly evaluate any clues that relate a given molecular alteration with human/animal/plant diseases or with novel curative/therapeutic interventions. Articles on the benefits of traditional medicines are acceptable if the conclusions include links between these interventions and regulations of molecular events.

Authors should discuss the results and how they can be interpreted from the perspective of previous studies and of the working hypotheses. The findings and their implications should be discussed in the broadest context possible. Future research directions may also be highlighted.

## The Compromise of *CIMB*

*CIMB* assures respect to any paper submitted to the journal, and decisions will be communicated as quickly as possible and will provide an accurate feedback. The Editorial and the Production teams will work together to guarantee a proper description of methods, results, but also proper sentence construction, i.e., accepted papers will not be published unless the style and the meaning of the sentences are clear. We are aware that ambiguity is common in many journals, including *CIMB*, but the journal’s commitment is firm on this specific (and relevant) topic.
